# Comparative Transcriptome Analysis of Sexual Differentiation in Male and Female Gonads of Nao-Zhou Stock Large Yellow Croaker (*Larimichthys crocea*)

**DOI:** 10.3390/ani14223261

**Published:** 2024-11-13

**Authors:** Haojie Wang, Zirui Wen, Eric Amenyogbe, Jinghui Jin, Yi Lu, Zhongliang Wang, Jiansheng Huang

**Affiliations:** 1 Fishery College, Guangdong Ocean University, Zhanjiang 524025, China; wanghaojie2@stu.gdou.edu.cn (H.W.); wenzirui@stu.gdou.edu.cn (Z.W.); 2112201133@stu.gdou.edu.cn (J.J.); hw402865@gmail.com (Y.L.); leong2006@126.com (Z.W.); 2 Department of Water Resources and Aquaculture Management, University of Environment and Sustainable Development, PMB, Somanya, Ghana; eamenyogbe@uesd.edu.gh; 3 Guangdong Provincial Key Laboratory of Aquatic Animal Disease Control and Healthy Culture, Zhanjiang 524088, China; 4 Guangdong Marine Fish Science and Technology Innovation Center, Zhanjiang 524088, China

**Keywords:** *Larimichthys crocea*, gonadal transcriptome, sex-associated genes, protein interaction network

## Abstract

The Nao-zhou stock large yellow croaker (*Larimichthys crocea*) is a unique economic seawater fish species in China and exhibits significant dimorphism in both male and female phenotypes. Cultivating all-female seedlings can significantly improve breeding efficiency. To accelerate the cultivation process of all female seedlings of this species, it is necessary to deeply understand the regulatory mechanisms of sexual differentiation and gonadal development. This study used Illumina high-throughput sequencing to sequence the transcriptome of the testes and ovaries of Nao-zhou stock large yellow croaker to identify genes and molecular functions related to sex determination. A total of 10,536 differentially expressed genes were identified between males and females, including 5682 upregulated and 4854 downregulated genes. Functional annotation screened out 70 important candidate genes related to sex, including 34 genes highly expressed in the testis (including *dmrt1*, *foxm1*, and *amh*) and 36 genes highly expressed in the ovary (including *gdf9*, *hsd3b1*, and *sox19b*). Gene Ontology (GO) and Kyoto Encyclopedia of Genes and Genomes (KEGG) pathway enrichment analysis found that differentially expressed genes were significantly enriched in nine signaling pathways related to sex determination and gonadal development, including steroid hormone biosynthesis, MAPK signaling pathway, and the TGF-beta signaling pathway. By screening sex-related differentially expressed genes and mapping protein–protein interaction networks, hub genes such as *dmrt1*, *amh*, and *cyp19a1a* were found to be highly connected. The expression levels of 15 sex-related genes, including *amh*, *dmrt1*, *dmrt2a*, *foxl1*, and *zp3b*, were determined by qRT–PCR and RNA sequencing. This study screened for differentially expressed genes related to sex determination and differentiation of Nao-zhou stock large yellow croaker and revealed the signaling pathways involved in gonad development of male and female individuals. The results provide important data for future research on sex determination and differentiation mechanisms, thereby providing a scientific basis for the cultivation of all-female seedlings.

## Simple Summary

This study focuses on the molecular mechanisms behind sexual differentiation in the Nao-zhou stock of large yellow croaker (*Larimichthys crocea*), a key marine species in China’s fisheries. By using high-throughput RNA sequencing to perform a comparative transcriptome analysis of male and female gonads, the research identifies critical genes and pathways involved in gonadal development. These findings advance our understanding of the genetic basis of sex differentiation, revealing specific molecular markers and regulatory networks that could guide breeding programs and improve understanding of reproductive biology in this commercially valuable species.

## 1. Introduction

Sexual differentiation is a fundamental biological process that underpins the reproductive success and the sustainability of fish populations [1,2]. Sexual dimorphism in fish includes individual size, morphological, and color dimorphism, as well as physiological and behavioral differences [3,4,5]. Sexual size dimorphism (SSD) has an obvious phenotype and is found in a variety of fish species [6,7,8]. Some economic fish species such as *Lepomis macrochirus* [9], *Tachysurus fulvidraco* [10], *Oreochromis mossambicus* [11], and *Odontobutis potamophilus* [12] have significantly faster growth rates in males than females, while in other species, including *Cyprinus Carpio* [13], *Cynoglossus semilaevis* [14], *Scatophagus argus* [6], and *Rachycentron canadum* [15], females have the higher growth rates. In fish with SSD, controlling the sex ratio of populations or even producing single-sex populations can improve aquaculture production and economic benefits [6,14,16,17]. Sex-related genes and their functions are constantly being discovered in fish, such as doublesex and mab-3-related transcription factor 1 (*dmrt1*) [18], gonadal soma-derived growth factor on the Y chromosome (*gsdfy*) [19], DEAD box polypeptide 4 (*vasa*) [20], cytochrome P450 family 19 subfamily A (*cyp19a*) [21], and anti-Mullerian hormone receptor 2 (*amhr2)* [22].

Transcriptome sequencing is one of the most powerful and effective methods for discovering functional genes and genetic markers. It can comprehensively and rapidly obtain almost all transcript sequence information from a specific tissue or organ in a certain state [6,14,16,17]. Gonads are crucial reproductive organs, and their development is usually controlled by multiple sex-related genes and pathways. Using transcriptome analysis of male and female glands, numerous differentially expressed genes (DEGs) between males and females were screened out, and a variety of key fish sex genes and the related signaling pathways were explored, laying the foundation for determining fish reproductive development and related mechanisms [23,24]. Transcriptome sequencing has been used to analyze gene expression in tilapia gonads at different stages and link gene expression and sexual differentiation with gonad development, creating a dynamic network of developmental stages and gene expression [25]. Multiple DEGs involved in gonad differentiation and gametogenesis were screened based on gene expression in late gonad development, and a weighted gene correlation network analysis (WGCNA) was performed. Genes related to known sex differentiation genes (*foxl2*, *cyp19a1*, *gsdf*, *dmrt1*, *amh*) were identified, including *borealin*, *gtsf1*, *tesk1*, *zar1*, *cdn15*, and *rpl* [25]. Transcriptome analysis of *Clarias fuscus* gonads revealed 5750 highly expressed DEGs in testes and 6991 highly expressed DEGs in ovaries that were enriched in sex-related pathways such as oocyte maturation, androgen secretion, gonadal development, and steroid biosynthesis [26]. By comparing the DEGs from body, ovarian, and testis tissues of largemouth bass (*Micropterus salmoides*), 22 crucial sex genes were identified. Among them, expression of the testis-specific *dmrt1* gene was correlated with highly expressed DEGs in the testis (*cyp11b1* and *spata4*) and the ovary (*foxl2*, *gdf9*, *zp3*, *sox3*, *cyp19a*, and *bmp15*). Analysis of the same genes in zebrafish showed that *dmrt1* was conserved and species-specific in sexual development [27].

The large yellow croaker, *Larimichthys crocea* (family Sciaenidae), is a commercially valuable marine species in China that has received significant attention due to its declining natural populations and the burgeoning aquaculture industry aimed at its conservation and commercial exploitation. Understanding the molecular mechanisms that drive sexual differentiation in this species is crucial for the development of effective breeding programs and maintaining genetic diversity. This species exhibits pronounced sexual dimorphism, particularly in its gonadal development, orchestrated by complex genetic and environmental interactions [28]. Advances in high-throughput sequencing technologies, such as RNA sequencing (RNA-seq), offer unprecedented opportunities to elucidate the transcriptomic mechanisms of gonadal development and sexual differentiation in this species [6,14,16,17]. Previous studies on fish have identified numerous genes and pathways involved in gonadal differentiation and development, including those related to hormone signaling, cell proliferation, and differentiation [1,3,29]. However, the specific transcriptomic profiles of male and female gonads in *L. crocea*, especially within the Nao-zhou stock, have not been investigated. This stock is particularly valuable due to its genetic distinctiveness and adaptation to local environmental conditions, making it an ideal candidate for detailed molecular studies. Therefore, in this study, high-throughput sequencing was used to analyze gene expression in the ovaries and testes of Nao-zhou stock large yellow croaker, identify DEGs related to sex determination and differentiation, and reveal the signaling pathways involved in gonad development of male and female Nao-zhou stock large yellow croaker. Furthermore, comparative transcriptomics determined the differential gene expression patterns in male and female gonads of this species. Using RNA-seq, this study aimed to identify the key regulatory genes and pathways that govern sexual differentiation in this species. In addition, the temporal expression profiles of potential genes involved in gonadal sex differentiation were measured using quantitative real-time reverse transcription PCR (qRT–PCR). These findings not only enhance our understanding of the molecular basis of sexual differentiation in *L. crocea* but also provide valuable insights for the aquaculture industry in terms of selective breeding and stock management. Specifically, the following objectives were addressed: to characterize the overall transcriptomic profiles of male and female gonads, to identify DEGs that are critical for gonadal differentiation, and to elucidate the biological pathways and molecular functions associated with these DEGs. By integrating bioinformatics with functional annotation, the study aimed to construct a comprehensive framework of the genetic architecture underlying sexual differentiation in Nao-zhou stock large yellow croaker. This research represents great progress in marine biology and aquaculture, contributing to the sustainable management and conservation of *L. crocea*. The results can inform breeding strategies and enhance our ability to maintain the genetic health of both wild and cultured populations of this economically and ecologically important species. Furthermore, this study also contributes important data for future research on the mechanisms of sex determination and differentiation, providing theoretical support for the cultivation of all-female Nao-zhou stock large yellow croaker.

## 2. Materials and Methods

### 2.1. Experimental Fish Sampling

Fish used in this experiment were collected from the waters of Naozhou Island, Zhanjiang City, Guangdong Province, China (N 20°41.404′, E 110°34.547′). The fish were anesthetized with 10 mg/L Tricaine methanesulfonate (MS-222) (Sigma Aldrich, St. Louis, MO, USA), and the testes and ovarian tissues were fixed with 4% paraformaldehyde and preserved in RNAlater (VivaCell, Shanghai XP Biomed Ltd., Shanghai, China) for analysis of gonadal histological characteristics (MG-1, FG-1), total RNA extraction (MG-1, MG-2, MG-3, FG-1, FG-2, FG-3), and sequencing (MG-1, MG-2, MG-3, FG-1, FG-2, FG-3). The average length of three male fish was 21.05 ± 0.30 cm, with an average weight of 76.67 ± 3.11 g, while the average length of three female fish was 29.30 ± 0.45 cm, with an average weight of 254.5 ± 4.72 g. All individuals of fish were labelled and listed with information about its sex and its application, such as it was used for histological analysis. The sampling procedure was approved by the Institutional Animal Ethics and Use Committee of Guangdong Ocean University (GDOU-LAE-2023-054).

### 2.2. Gonad Tissue Sections

Histology was used to clarify the gonad development stage of the samples. Gonad tissue was preserved in 4% paraformaldehyde, fixed according to the size of the gonads for 2–6 h, and transferred to 70% ethanol for long-term storage. Paraffin sections were processed by Bouin’s fixative, ethanol gradient dehydration, xylene permeabilization, paraffin immersion, and embedding. The paraffin samples were serially sectioned with a thickness of 4 μm, stained with hematoxylin–eosin (Biosharp, Beijing Labgic Technology Co., Ltd., Beijing, China), and mounted with neutral resin (Biosharp, Beijing Labgic Technology Co., Ltd., Beijing, China). The morphology and structure of gonads were observed and photographed under an upright microscope (Leica, Wetzlar, Germany).

### 2.3. RNA Extraction and Detection

Following the methods described by Yang et al. [30], in our study, total RNA was extracted from the intestinal tissues of Nao-zhou stock large yellow croaker using TRIzol reagent (Invitrogen, Carlsbad, CA, USA). The purity and concentration of extracted RNA were detected using a nanophotometer, a spectrophotometer (Thermo Fisher, Waltham, MA, USA), and a qubit2.0 fluorometer (Invitrogen, Carlsbad, CA, USA). Agarose gel electrophoresis and a bioanalyzer (Agilent 2100, Agilent Technologies, Santa Clara, CA, USA) were used to assess the integrity and detect contamination in extracted RNA. Select RNA samples with an A260/A280 ratio between 1.8 and 2.0 and an agarose gel electrophoresis band with a 28S:18SrRNA ratio close to 2:1 were selected and stored at −80 °C until use.

### 2.4. Transcriptome Library Construction and Sequencing

After ensuring the quality of RNA, mRNA was enriched using magnetic beads containing Oligo (dT). The mRNA was randomly broken using ultrasound, and then single-stranded cDNA was synthesized using fragmented mRNA as a template. RNA strands were degraded using RNaseH and double-stranded cDNA was synthesized using dNTPs as raw materials in the DNA polymerase I system. After purification, the synthesized double-stranded cDNA was first repaired at the end, followed by adding an A-tail and connecting sequencing adapters. Then, AMPure XP beads were used to screen for cDNA fragments of approximately 200 bp for PCR amplification and further purification. Finally, three cDNA libraries of male and female Nao-zhou stock large yellow croaker were constructed. The library was sequenced on an Illumina NovaSeq 6000 machine, and the library construction and transcriptome sequencing were completed by Gene Denovo Biotechnology Co., Ltd. (Guangzhou, China) [30].

### 2.5. Transcriptome Assembly and Annotation

To ensure the quality of data for subsequent analysis, the original reads containing adapters or low-quality bases from the sequencing machine were filtered to obtain high-quality clean reads. First, fastp (version 0.18.0) was used to screen reads, remove reads containing adapters, reads with N content exceeding 10%, reads containing over 50% low-quality information (*Q* value ≤ 20), reads containing all A bases, and contaminated reads. Then, Bowtie2 (version 2.2.8) was used to compare the obtained reads with the ribosomal RNA (rRNA) database to remove mapping reads [30]. Finally, HISAT2 was used to pair the clean reads with the reference genome of Nao-zhou stock large yellow croaker for subsequent annotation.

### 2.6. Gene Expression Levels and Differential Enrichment Analysis

StringTie v1.3.1 and RSEM v1.3.3 software were used to assemble the mapped reads of each sample and calculate the fragments per kilobase of exon model per million mapped fragments (FPKM) to quantify gene expression abundance. Expression information was then analyzed by principal component analysis (PCA) and correlation analysis (Pearson correlation analysis) between samples and varieties. DEseq2 1.1.0 [31] software was used for standardization and differential expression gene detection, with differential fold |log_2_ fold change| ≥ 1 and false discovery rate (FDR) ≤ 0.05 as the gene screening thresholds. DEGs between male and female gland groups were screened out (ovary group: FG-1, FG-2, FG-3; testis group: MG-1, MG-2, MG-3). Gene Ontology (GO) functional annotation and Kyoto Encyclopedia of Genes and Genomes (KEGG) pathway enrichment were performed on DEGs.

### 2.7. Protein-Protein Interaction Network (PPI) Analysis of Key DEGs Between Sexes

The STRING database and Cytoscape software were used to conduct PPI analysis of key DEGs between sexes [32]. Blastx was used to align the sequences in the target gene set to the reference species protein sequences included in the STRING database (www.string-db.org, accessed on 1 June 2024), and the aligned protein interaction relationships of the reference species were used to construct an interaction network. The points in the PPI network diagram are genes and the lines represent the interaction between proteins (genes) and proteins (genes).

### 2.8. RT-qPCR 

Primer Premier 5 software was used to design specific primers for DEGs related to gonad development (Table 1), which were synthesized by Sangon Bioengineering (Shanghai, China) Co., Ltd. First, 1 μg of RNA from each of the six sequenced gonads was used as a template and reverse transcribed to cDNA according to the instructions of the PrimeScript RT reagent Kit with gDNA Eraser (Takara, Japan). *β-actin* was used as an internal reference gene, and RT-qPCR was used to verify 15 sex-related genes. RT-qPCR was carried out on the ABI QuantStudio6 FLEX Q6 real-time fluorescence quantitative PCR instrument (Applied Biosystems, Waltham, MA, USA) according to the instructions of the PowerUpTM SYBRTM Green Master Mix kit (Applied Biosystems, Waltham, MA, USA). The reaction program was as follows: predenaturation at 50 °C for 2 min, 95 °C for 10 min, three-step amplification for 40 cycles, 95 °C for 15s, and reaction at 58 °C for 15s. The melting curve was 95 °C for 15 s, 60 °C for 60 s, and 95 °C for 15 s. The samples were run with three technical replicates, and the relative expression of genes was analyzed using the 2^−ΔΔCt^ method [33].

## 3. Results

### 3.1. Histological Characteristics of the Gonads of Nao-Zhou Stock Large Yellow Croaker

**Figure 1:**
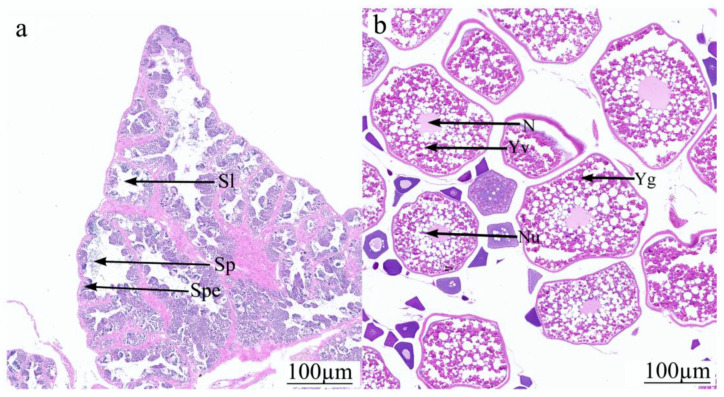
Histological characteristics of testes (**a**) and ovaries (**b**) of Nao-zhou stock large yellow croaker. Notes: Sp: sperm; Spe: sperm cell; Sl: sperm lobule; Yg: yolk granule; N: nucleus; Yv: yolk vesicle; Nu: Nucleolus.

Histology showed that the gonad samples from the testis and the ovary groups had the same development period. Testis samples (MG-1, MG-2, MG-3) developed to stage IV. At this stage, the seminiferous tubules were composed of primary spermatocytes, secondary spermatocytes, and a small number of sperm (Figure 1a). The testis is characterized by the presence of primary spermatocytes, secondary spermatocytes, and a small number of seminiferous tubules. The primary spermatocytes undergo meiosis to form secondary spermatocytes, which further divide to produce spermatids and eventually mature into seminiferous tubules. Ovarian samples (FG-1, FG-2, FG-3) developed to stage IV. The yolk granules and yolk vesicles fill the extranuclear space and are stained red in the vicinity of the nucleus, showing a large ring (Figure 1b). At this stage, the ovarian follicles are characterized by the presence of yolk granules and yolk vesicles, which fill the extranuclear space. These yolk materials are essential for providing nutrients to the developing oocytes.

### 3.2. Transcriptome Results and Quality

Three high-throughput sequencing libraries for stage IV testes and stage IV ovaries of Nao-zhou stock large yellow croaker were constructed. High-throughput sequencing obtained a total of 38,332,782,600 fragments of raw data, with 37,939,156,295 clean reads at a ratio of 98.97%. After filtering, the proportion of Q20 bases exceeded 98%, the proportion of Q30 bases exceeded 95%, and the GC content accounted for 50.40~51.02%. The data integrity was high and suitable for subsequent analysis (Supplementary Table S1).

The clean reads of the six samples were aligned to the reference genome sequence (Supplementary Table S2). The average total number of effective sequencing data reads for ovarian and testicular samples were 42,349,865 and 42,138,256, respectively, and the total sample comparison ratios were 85.29% and 85.47%, respectively. A hierarchical alignment strategy was further adopted to align reads and splice reads of different lengths to the reference genome. An average of 79.61% of the sample genome reads were located in the exon region. The gene annotation and reference genome were relatively complete and could be used for subsequent analysis.

### 3.3. Differential Gene Identification and Enrichment Analysis

**Figure 2:**
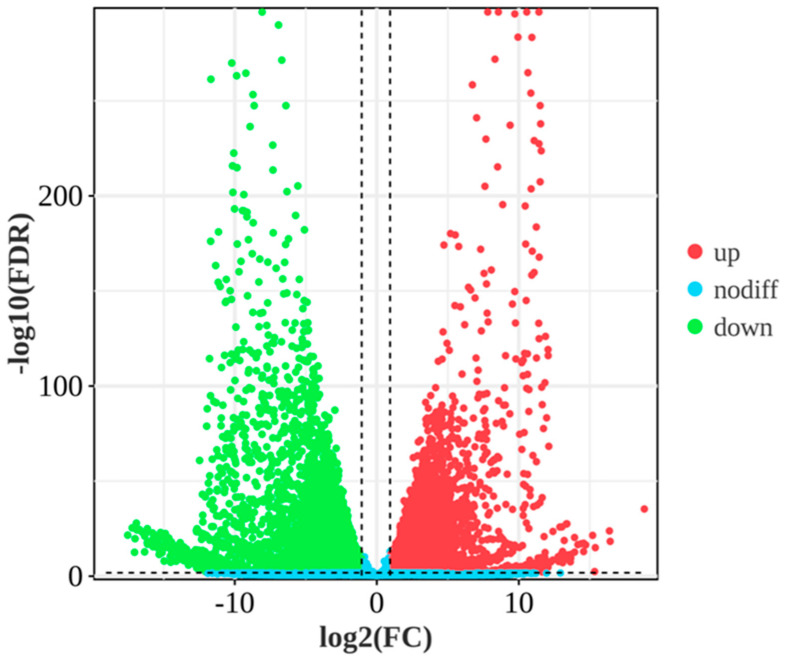
Volcano map of differentially expressed genes in Nao-zhou stock large yellow croaker. Note: The horizontal axis shows the log_2_ value (fold change), the vertical axis is the −log_10_ value (*p* value), green dots represent upregulated genes, red dots represent downregulated genes, and blue dots represent genes with no significance. The dotted lines represent the threshold of log_2_(FC) values.

**Figure 3:**
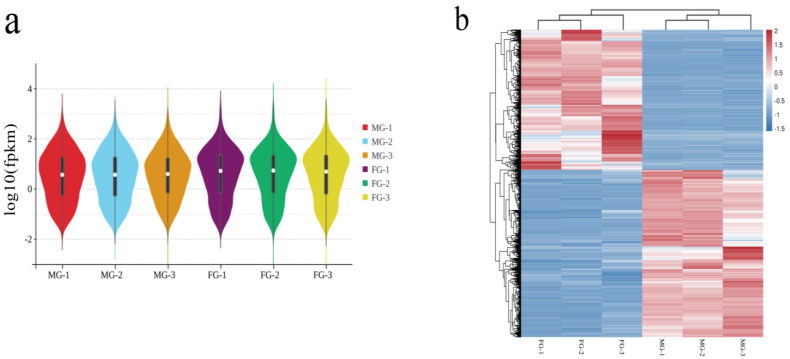
Violin plot and cluster heat map of 6 samples. Note: (**a**) represents the correlation of samples between and within groups. (**b**) shows cluster results of DEGs. The color indicates the expression amount (logarithm) or the difference multiple (logarithm). The redder color indicates that the gene expression level is higher or the difference factor is larger, and the blue color indicates the opposite.

To determine the molecular mechanism of gonadal development in this species, the gene expression levels of male and female specimens were compared. Based on the expression index FPKM, the expression of the same gene was analyzed, with *p* < 0.05 and |log_2_(fold change)| > 1 as the threshold. A total of 10,536 DEGs were found in testis and ovary tissues (Figure 2). Taking testis tissue as the control, 5682 DEGs were upregulated and 4854 DEGs were downregulated in the ovary. Further analysis of DEGs between males and females via violin plot (Figure 3a) showed that the composition of samples within the ovary and testis groups was similar and highly correlated, while the composition of samples between groups was low and the correlation coefficient was extremely low. This indicates that there were large differences in gene expression between males and females, while the differences within the groups were small. A hierarchical cluster analysis of all samples was performed based on gene expression information (Figure 3b) and showed that the ovary and testis samples were clustered into one branch, which was consistent with the correlation analysis.

**Figure 4:**
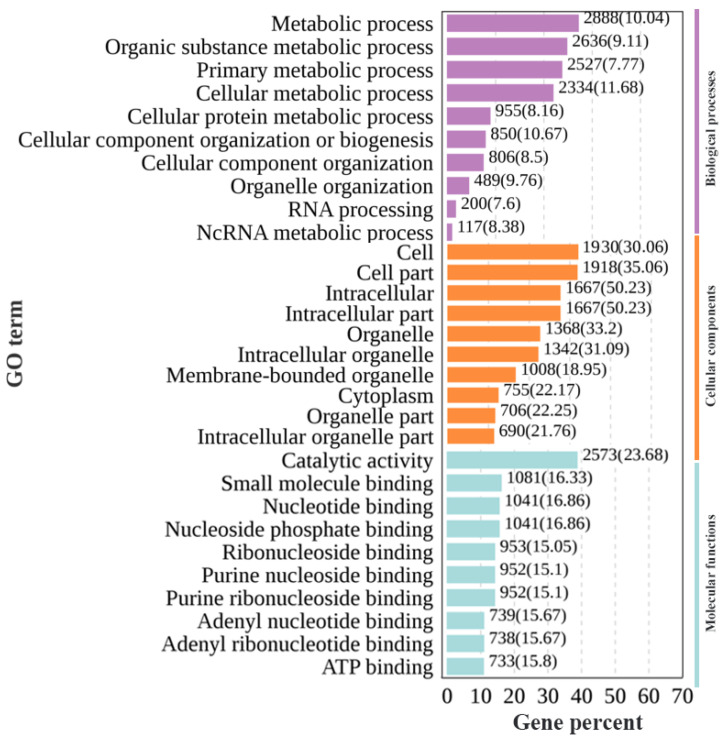
Top 30 GO enrichment pathways of differentially expressed genes in the gonads of Nao-zhou stock large yellow croaker. Note: The horizontal axis shows the gene name, and the vertical axis shows the gene ratio.

To explore the distribution of DEGs through GO analysis and the biological processes they participate in within the gonads, 10,536 DEGs were annotated into 727 GO branches, which were mainly divided into three categories: biological processes, cellular components, and molecular functions (Figure 4). There were 436 annotations in biological processes, and the top three DEG annotations were primary metabolic process (GO:0044238), organic substance metabolic process (GO:0071704), and metabolic processes (GO:0008152); there were 133 annotations in cellular components, and the top three DEG annotations were cells (GO:0005623), cell parts (GO:0044464), and intracellular (GO:0005622); finally, there were 158 annotations in molecular functions, and the top three DEG annotations were small molecule binding (GO:0036094), catalytic activity (GO:0003824), and nucleotide binding (GO:0000166).

**Figure 5:**
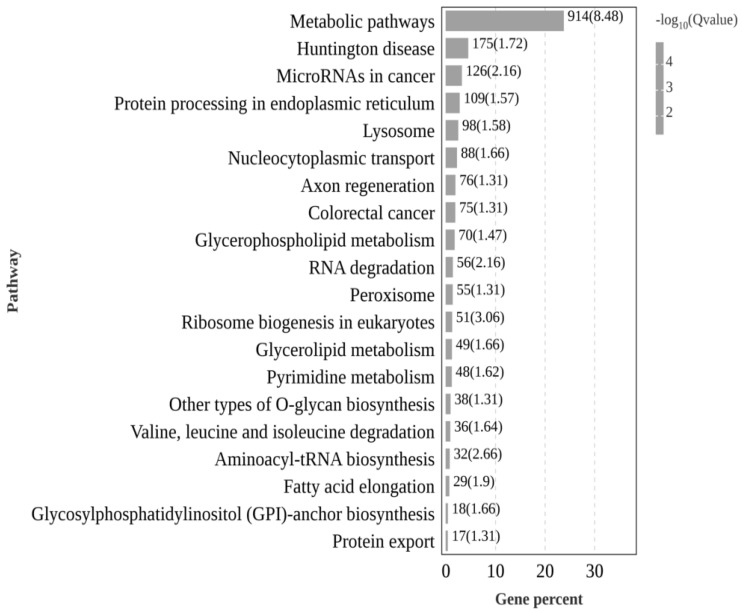
Top 30 KEGG enrichment pathways of differentially expressed genes in the gonads of Nao-zhou stock large yellow croaker. Note: The horizontal axis shows the gene name, and the vertical axis shows the gene ratio.

To further identify the specific functions of DEGs in the gonads, the enriched signal pathways were further analyzed using the KEGG database (Figure 5). A total of 10,536 DEGs were enriched in 64 signal pathway categories. The enrichment information of metabolic pathways of the top 30 DEGs is shown in Figure 5. The DEGs with the highest annotation ratio were metabolic pathways (Ko:01100), such as protein processing in the endoplasmic reticulum (Ko:04141) and aminoacyl-tRNA biosynthesis (Ko:00970), which are related to growth and reproduction. 

### 3.4. Predicted Function of the Sex-Biased Genes

Seventy sex-related genes were analyzed that play a vital role in sex determination and gonad development in other vertebrates. Among them, 34 genes were significantly expressed in the testis and 36 genes were highly expressed in the ovary; these are presented according to their log_2_ fold change (FC) values in Table 2.

**Figure 6:**
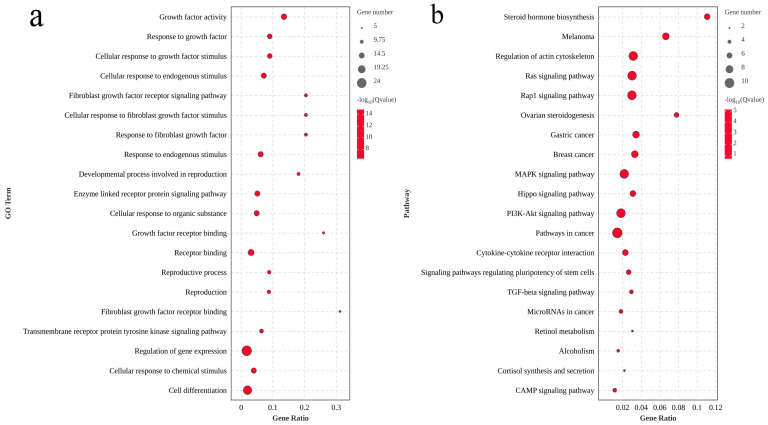
GO (**a**) and KEGG (**b**) enriched pathways of the top 20 differentially expressed genes associated with sex in Nao-zhou stock large yellow croaker.

GO and KEGG enrichment analyses were performed on these genes (Figure 6a,b). GO annotation showed that reproduction (GO:0000003), reproductive process (GO:0022414), and growth factor activity (GO:0008083) were related to gonad development and reproduction in Nao-zhou stock large yellow croaker. KEGG pathway analysis identified that steroid hormone biosynthesis (ko:00140), the MAPK signaling pathway (ko:04010), and the TGF-beta signaling pathway (ko:04350) were also involved. These results indicate that these pathways play a key role in the gonad development of Nao-zhou stock large yellow croaker.

### 3.5. PPI Network Analysis of Key DEGs Between Sexes

**Figure 7:**
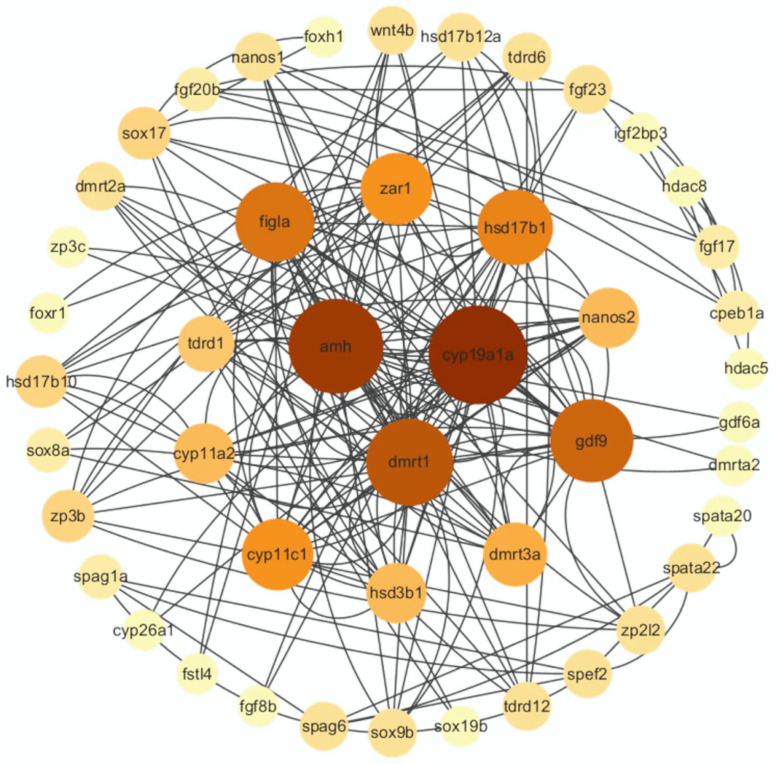
Protein-protein interaction (PPI) network diagram of DEGs in female and male Nao-zhou stock large yellow croaker. Note: Different background colors represent the network degree values of proteins. The inner circle of the PPI network shows hub genes, while the outer two circles are non-hub genes. Number of gene nodes is represented by color depth.

To explore the correlation of these DEGs, a PPI network analysis of 46 sex-related DEGs was performed using the STRING database and Cytoscape software (Figure 7). A network diagram of 46 nodes and 198 protein interaction pairs was constructed, including sex-related genes such as *dmrt1*, *foxr1*, *amh*, *sox19b*, and *cyp11a2*. Among them, *dmrt1*, *amh*, and *cyp19a1a* were identified as hub genes.

### 3.6. qRT-PCR Validation of Differential Sex Expression of Genes

**Figure 8:**
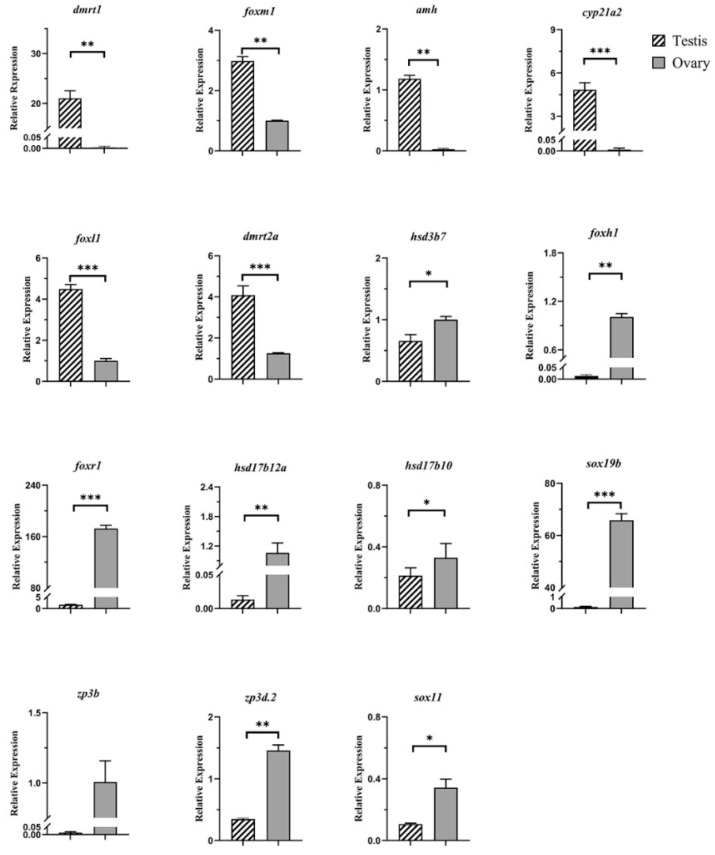
Relative expression levels of 15 genes in the testis and ovary of Nao-zhou stock large yellow croaker. Note: Data are presented as mean ± S.E.M. (n = 3). The asterisks indicate that the differences between the mean values are statistically significant between gonads. *: 0.01 < *p* < 0.05; **: 0.001 < *p* < 0.01; ***: *p* < 0.001.

**Figure 9:**
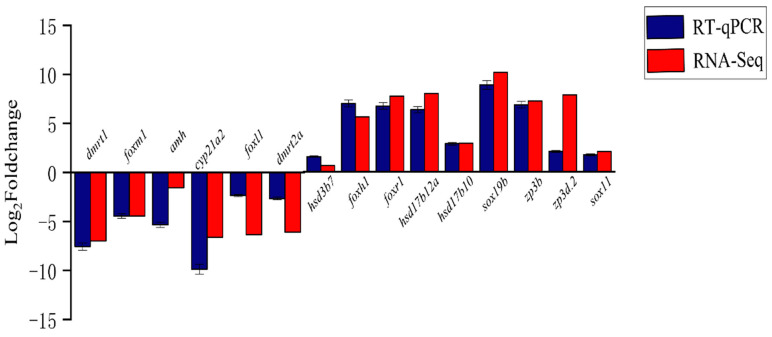
qRT-PCR verification of sex-related differentially expressed genes. Note: The horizontal axis shows the gene name, and the vertical axis shows the relative expression level.

**Figure 10:**
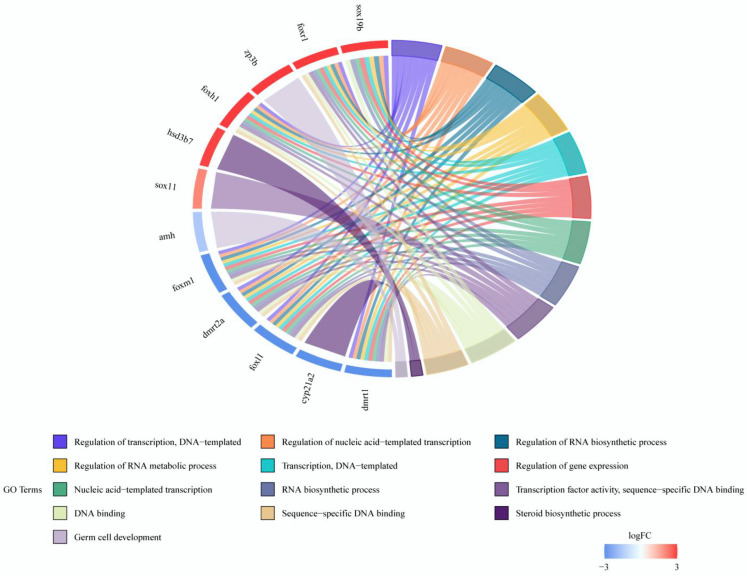
Chord diagram of the functional classification of twelve candidate genes. Note: The left half represents candidate genes and expression levels, and the right half represents GO enriched pathways related to reproduction.

To validate the differential sex expression of genes, 15 sex-biased DEGs were randomly selected, and their relative expression in the testis and ovary was analyzed (Figure 8). The relative expression of *dmrt1*, *foxm1*, *amh*, *cyp21a2*, *foxl1*, and *dmrt2a* in the testis was significantly higher than in the ovary, while the expression of *hsd3b7*, *foxh1*, *foxr1*, *hsd17b12a*, *hsd17b10*, *sox19b*, *zp3b*, *zp3d.2*, and *sox11* was higher in the ovary (all *p* < 0.05). The expression pattern detected by qRT-PCR is consistent with that from RNA-seq differential analysis (Figure 9), demonstrating the accuracy of the RNA-seq analysis. The chord diagram (Figure 10) shows that sex candidate genes such as *foxr1*, *dmrt2a*, *sox19b*, *foxh1*, and other genes were involved in most gonadal development and reproductive processes.

## 4. Discussion

### 4.1. Histological Analysis of Gonad Development in Testis and Ovary of Nao-Zhou Stock Large Yellow Croaker (Larimichthys crocea)

Despite its economic importance, the molecular mechanisms of sexual differentiation in *L. crocea* remain poorly understood. Previous studies have identified three geographical populations of large yellow croaker: the Dai-qu stock, Min-yuedong stock, and Nao-zhou stock, from north to south [31]. Nao-zhou stock large yellow croaker is an indigenous population in the South China Sea that has phenotypic characteristics that are obviously different from the large yellow croaker population on the eastern coast of China [34]. Compared with Min-yuedong and Dai-qu stock large yellow croaker, Nao-zhou stock large yellow croaker has more unique traits and qualities of wild large yellow croaker, as well as greater genetic diversity. Owing to the obvious sexual dimorphism in Nao-zhou stock large yellow croaker, females grow much faster than males. Therefore, cultivating all-female individuals can improve the efficiency of aquaculture. However, there are no reports on the gonadal development and related regulatory mechanisms of Nao-zhou stock large yellow croaker. 

Histology of gonad samples from both testis and ovary of Nao-zhou stock large yellow croaker revealed insights into their developmental stages. The analysis aimed to compare the maturation process of male and female gonads to determine whether they follow a similar developmental timeline and to elucidate the specific cellular changes that occur during these stages. The testis samples (MG-1, MG-2, and MG-3) all progressed to stage IV of development (Figure 1a). At this stage, the testis is characterized by the presence of primary spermatocytes, secondary spermatocytes, and a small number of seminiferous tubules. The primary spermatocytes undergo meiosis to form secondary spermatocytes, which further divide to produce spermatids and eventually mature into seminiferous tubules [35,36]. The presence of these cell types indicates active spermatogenesis, reflecting the role of the testis in producing male gametes [37,38,39]. Histologically, this stage is marked by densely packed cells within the seminiferous tubules, where the different stages of sperm development can be observed.

Similarly, the ovarian samples (FG-1, FG-2, and FG-3) also developed to stage IV (Figure 1b). At this stage, the ovarian follicles are characterized by the presence of yolk granules and yolk vesicles, which fill the extranuclear space. These yolk materials are essential for providing nutrients to the developing oocytes [40,41,42]. Histological staining showed these granules stained red near the nucleus, forming a large ring. This indicates that the oocytes are accumulating yolk, a process known as vitellogenesis, which is crucial for the maturation of the eggs and their readiness for fertilization. The parallel development to stage IV in both testis and ovary samples suggests a synchronized maturation process, where both male and female gonads reach a comparable stage of readiness for reproduction. The histological characteristics observed in both groups highlight the specific functions of these organs in gamete production. In the testis, the progression through various stages of spermatogenesis ensures the continuous production of spermatozoa, necessary for fertilization. The presence of primary and secondary spermatocytes, along with spermatids, indicates a robust spermatogenic activity. In contrast, ovarian development focuses on the preparation of oocytes for potential fertilization. The accumulation of yolk granules and vesicles provides the necessary nutrients for the oocytes, ensuring they are well equipped for the early stages of embryonic development postfertilization.

Histology of gonad samples from both testis and ovary of Nao-zhou stock large yellow croaker demonstrates that both groups reach stage IV of development, albeit with distinct cellular characteristics pertinent to their roles in reproduction. The testis samples showed active spermatogenesis with the presence of various stages of sperm cells, while the ovarian samples exhibited advanced vitellogenesis, marked by the accumulation of yolk materials around the oocytes. These findings indicate a synchronized developmental timeline between male and female gonads of Nao-zhou stock large yellow croaker, ensuring that both are prepared for the reproductive process simultaneously. This synchronization is critical for successful fertilization and subsequent embryonic development. The study provides valuable insights into the reproductive biology of the Nao-zhou stock large yellow croaker, contributing to our understanding of gonadal development and its implications for fertility and reproduction.

### 4.2. DEGs Related to Male and Female Gonadal Reproduction in Nao-Zhou Stock Large Yellow Croaker

Among the genes differentially expressed between male and female gonads of Nao-zhou stock large yellow croaker, 70 sex-critical genes, including *dmrt1*, *foxh1*, *sox19b*, *zp3b*, and *hsd17b10*, were further screened as candidate genes for sex determination. Sex determining genes are an important regulatory factor that controls sexual differentiation and have been the focus of widespread attention. Fish sex-determining genes discovered so far can be divided into three major categories, which provide clues for studying sex-determining genes in other fish: transcription factors, TGF-β family-related genes, and steroid hormone synthesis-related genes.

The transcription factor *dmrt1* was first discovered in invertebrates and plays an important role in the sex determination and differentiation process of fish [43]. By knocking out the *foxl2* and *dmrt1* genes in Nile tilapia using TALEN, male testes lacking *dmrt1* and spermatogonia degenerated, and even germ cells were completely missing [44]. Webster et al. found that zebrafish (*Danio rerio*) *dmrt1* mutants developed into fertile females and infertile males. The loss of *dmrt1* had different effects on males and females: females developed normally, while male gonads differentiated abnormally and could not differentiate into testicular structures and produce sperm. These results suggest that *dmrt1* plays an important role in the transition period from ovary to testis in zebrafish larvae and in the development of male germ cells [45]. Similar results were found in *dmrt1* mutants of Chinese tongue sole (*Cynoglossus semilaevis*) [46] and medaka (*Oryzias latipes*) [47], which showed impaired testicular development. Studies on the expression of *dmrt1* in the gonads of various fish species show that *dmrt1* is highly expressed or specifically expressed in the testes, such as in Japanese flounder (*Paralichthys olivaceus*) [48] and Tiger pufferfish (*Takifugu rubripes*) [49]. The expression of *dmrt1* in testicular tissue was significantly higher than in ovarian tissue, indicating that *dmrt1* is essential during testicular development. In this study, sequencing the gonad transcriptome of Nao-zhou stock large yellow croaker annotated a total of two DM domain genes, both of which were highly expressed in the testis. Among them, there was a significant difference in the expression levels of *dmrt1* and *dmrt2a* in the testis and ovary *(p* < 0.05). The PPI network analysis showed that *dmrt1* had direct interactions with DEGs highly expressed in both the testis (*sox9b*, *nanos1*, *nanos2* and *amh*) and ovary (*gdf9*, *hsd3b*, *zar1*, *hsd17b1*, *cyp11a2* and *figla*), indicating that *dmrt1* may play a role in testis and ovary development by inducing the expression of testis-related genes and ovary-related genes.

Forkhead box (FOX) genes encode a family of evolutionarily conserved transcription factors that play important roles in a variety of biological processes, including metabolic and immune regulation, control of the cell cycle and cell survival, and sex determination and differentiation [50]. *Foxl1* is an important member of the forkhead transcription factor superfamily, which is specifically expressed in the granulosa cells of the vertebrate ovary and is involved in ovarian differentiation and oogenesis [51]. The FOX (forkhead box) gene family plays diverse roles in regulating growth, development, and differentiation processes [52,53]. Regarding female growth, certain FOX genes are known to be involved in signaling pathways that regulate sex-specific growth patterns, reproductive development, and metabolic processes [54,55]. In fish species, for example, FOX genes may influence growth by regulating key genes involved in hormone signaling and metabolism that are critical for female development [56,57,58]. Genes like FOXO, for instance, are linked to insulin signaling pathways, which can influence growth by modulating metabolic rate and energy storage [59]. Other FOX genes, such as FOXL2, are well-documented in roles related to ovarian development and function, which are essential for reproductive growth and development in females [60]. Thus, highlighting these connections could provide insight into how FOX genes support not only the growth of female individuals but also contribute to reproductive and overall metabolic processes that are specific to female growth trajectories. In this study, *foxl1* was highly expressed in the ovaries and therefore is related to the differentiation and development of female gonads. A total of 54 FOX domain genes were annotated by transcriptome sequencing of the gonads of the Nao-zhou stock large yellow croaker. Among them, *foxm1*, *foxo4*, and *foxl1* were highly expressed in males, while *foxh1*, *foxo3*, etc., were highly expressed in females. There were significant differences in the expression levels of these genes in the testes and ovaries (*p* < 0.05).

The amino acid sequences of SOX family members are highly conserved in the high mobility group (HMG)-box region and play an important role in the differentiation of testes and other tissues [61]. In this study, a total of 23 members of the SOX gene family were annotated, among which *sox9b*, *sox6*, *sox11*, *sox18*, and *sox7* were highly expressed in the testes, while *sox4a*, *sox19b*, *sox10*, and *sox17* were mainly expressed in the ovaries, suggesting that SOX family members play a complex role in the sexual differentiation and development of the study species. The *sox9* gene has previously been found to be mainly expressed in the testes during gonadal differentiation, and could promote the differentiation of testicular supporting cells and interstitial cells as well as testicular development. It is considered a key gene for sex determination and gonadal development in mammals [62]. The amino acid sequences of *sox9a* and *sox9b* of large yellow croaker were cloned, and the gene expression levels in different tissues and developmental stages were analyzed. The expression of *sox9a* and *sox9b* was highest in the testis, which was significantly higher than in the ovary and other tissues, while the expression of *sox9a/b* in the early stage of gonadal development was lower than in the later stage of development. This indicates that the expression of *sox9* is sexually dimorphic and may play an important role in gonadal development [63]. *sox9a* has been found to be highly expressed in ovarian tissue of medaka while *sox9b* was highly expressed in the testis [64]. In contrast, *sox9a* and *sox9b* were highly expressed in the testis and ovary of zebrafish [65], respectively. Therefore, it is necessary to study the expression pattern of *sox9* in different fish species. In addition to *sox9*, other SOX family members have been shown to be related to sex differentiation and gonad development. For example, *sox3* is the sex determination gene of medaka [66]; *sox4*, *sox5*, *sox6*, and *sox8* play a role in spermatogenesis; and *sox2* and *sox3* are involved in testis and ovary development, respectively [67,68]. In conclusion, the SOX gene family plays an important role in sex determination and gonadal differentiation and development.

Anti-Mullerian hormone (AMH) is a glycoprotein of the transforming growth factor-β (TGF-β) superfamily [69]. In Japanese flounder (*Paralichthys olivaceus*) [70] and rainbow trout (*Oncorhynchus mykiss*) [71], the expression of *amh* was significantly higher in testis than in ovary tissue. In zebrafish, *amh* regulates the accumulation of male germ cells and inhibits the development or survival of oocytes [72]. In Japanese eels (*Anguilla japonica*), *amh* was only expressed in male eels that had not reached sexual maturity and was not detected in females [73]. In this study, a total of four *amh* gene family members were annotated. The expression of *amh* in the testis was significantly higher than in the ovary, indicating that *amh* may be necessary for male sexual differentiation in Nao-zhou stock large yellow croaker. It was also possible that *dmrt1* may affect male sexual characteristics through the transcriptional regulation of *amh* [45].

The *hsd3b* gene family is thought to be involved in the steroidogenesis process in mammals, converting pregnenolone to progesterone, hydroxypregnenolone to 17α-hydroxyprogesterone, and androstenediol to testosterone [74]. Previous studies have shown that the *hsd3b1* gene could convert pregnenolone into progesterone, which was further converted into androstenedione in follicular cells [75]. In the testis of orange-spotted grouper (*Epinephelus coioides*), the expression of *hsd3b7* is higher than in the ovary [76]. In contrast, the expression of hsd3b7 is higher in the ovaries of pearlscale angelfish (*Centropyge vrolikii*) than in the testes and was significantly reduced during sexual transition [77]. After knocking out the *foxl2* gene in the ovary cell line of large yellow croaker through RNAi, the expression of *hsd3b7* increased significantly, while after knocking out the *dmrt1* gene in the testis cell line, the expression of *hsd3b7* was significantly reduced, indicating that both *foxl2* and *dmrt1* play an important role in the synthesis of *hsd3b7*. In this study, the expression of *hsd3b7* in the ovary of Nao-zhou stock large yellow croaker was significantly higher than in the testis. In addition, the PPI network showed that *hsd3b1* interacts with *cyp19a1a*, *figla*, and *dmrt1*, among others.

The zona pellucida (ZP) protein family is the main component of the transparent envelope surrounding teleost oocytes. ZP proteins play an important role in sperm-egg recognition, inducing acrosome reaction, oocyte maturation, preventing polyspermy, and fertilization [78].

Previous studies have shown that fish egg envelopes usually contain two to four ZP family genes, which are homologous to mammalian *zp1*, *zp3*, and *zp4* [79]. *zp2* plays an important role in the early formation of the oocyte envelope, while *zp3* participates in reproductive activities such as the acrosome reaction and is the main component protein of fish eggshells [80]. There is limited research on the fish *zp4* gene. Human *zp4* could induce the acrosome reaction and inhibit the binding of sperm to the ZP. In this study, the expression of two ZP family genes (*zp3b*, *zp3d.2*) in the ovaries of Nao-zhou stock large yellow croaker was significantly higher than in the testes, indicating that they play important roles in the folliculogenesis process.

### 4.3. Signaling Pathways Related to Gonadal Reproductive Regulation in Nao-Zhou Stock Yellow Croaker

In this study, GO and KEGG annotation analyses were used to obtain the functional information of a large number of DEGs in the gonads of male and female Nao-zhou stock large yellow croaker. The GO analysis identified sex differentiation, reproductive process, gamete formation, gonad development, steroid hormone biosynthesis, supporting cell differentiation, sperm binding to zona pellucida, retinoic acid receptor signaling pathway, and retinoic acid decomposition processes. Among them, sex differentiation, sexual reproduction, reproductive process, gamete formation, and gonad development related to specific sex-related genes were shown to play an important role in the sex determination and differentiation of fish and affect the subsequent spermatogenesis and oogenesis processes [81]. The functional annotations of DEGs indicate that they may play an important role in sex determination and differentiation, gamete formation, spermatogenesis, embryonic development, and other aspects of Nao-zhou stock large yellow croaker reproduction and participate in physiological processes related to the formation of sex dimorphism to produce functional effects. These results provided important information for subsequent research on marker genes for sex determination and differentiation, as well as sex control.

The KEGG annotation results also obtained rich information on the functional pathways of male and female gonads, including the PI3K-Akt signaling pathway, insulin signaling pathway, FoxO signaling pathway, and TNF signaling pathway. The PI3K-Akt signaling pathway has been identified as playing an important regulatory role in oocyte growth and early follicle development [82]. The insulin signaling pathway regulates physiological processes of development, metabolism, and lifespan and interacts with other pathways [83], while the PI3K-Akt signaling and insulin signaling pathways are involved in important processes of gonadal development and maturation [84]. Further screening revealed that the steroid hormone biosynthesis signaling pathway was involved in the biosynthesis process of sex hormones, and its synthetic steroid-related genes had a direct impact on the development or differentiation of gonads. In this study, there were significant differences in the expression of multiple steroid hormone synthases in this pathway with genes such as *cyp21a2*, *cyp19a1a*, *hsd11b1la*, *hsd17b8*, and *hsd17b7* being highly expressed in the testes, reflecting their important regulatory role in the normal reproduction of male and female individuals. The differential analysis of signal pathways provides a direction for the signal regulation involved in sexual dimorphism; the specific regulatory process still requires more in-depth research on the related genes involved in these pathways.

## 5. Conclusions

The development of gonads is essential for animal reproduction, involving key stages that include undifferentiated, differentiating, and differentiated phases. The genetic mechanisms are complex and involve specific genes that promote or sustain the development of gonads into either testes or ovaries. Transcriptomics offers a powerful approach to uncovering gene regulatory networks between males and females. This study on the Nao-zhou stock large yellow croaker (*Larimichthys crocea*) provides valuable insights into the molecular mechanisms of sex determination and gonadal differentiation in this species. High-throughput transcriptome sequencing identified 10,536 differentially expressed genes (DEGs) between male and female gonads, with significant enrichment in pathways related to reproduction, steroid hormone biosynthesis, and sex differentiation. Seventy key genes for sex determination, such as *dmrt1*, *spag6*, *foxl1*, *amh*, and *sox19b* in testes and *foxr1*, *gdf9*, *sox11*, *hsd3b1*, and *zp3d.2* in ovaries, were identified as crucial for gonadal development. Protein-protein interaction (PPI) analysis highlighted that *dmrt1* and *amh* play central roles in testis development, while other DEGs are critical for ovarian development, indicating distinct regulatory networks for male and female gonads. This study provides important data on sex-related and gonadal gene expression in this economically significant species and serves as a valuable resource for genomic studies and the development of sex control strategies.

## Figures and Tables

**Table 1:** Primers of the genes used for Quantitative Reverse Transcription Polymerase Chain Reaction (qRT-PCR).

Gene	Forward Primer 5′-3′	Reverse Primer 5′-3′
*dmrt1*	AACAGCAACAACAACAACAGCAACA	TCACACTCAGCGTGGACATCAGA
*foxm1*	TGTCGCCTCCTCGGTCTATCTG	ACTCTGGCTCGTCCTTCACCTT
*amh*	GTCTTCTGCTGTGGAACGCTGAT	GCTTGTTGTCTGGTGGTCTCCTT
*cyp21a2*	ACAGAGCCATCAGAGACAGCAGTA	CAGCAAGTAGGCGGTGAACAGAA
*foxl1*	ACTCCATCCGCCACAACCTCT	GCTCCTTGCCTTCCTCTTCCTC
*dmrt2a*	GACGGCAGTAGAAGTGACGACAAG	TCCACCACCAGCAGGCAGTT
*sox19b*	CAGAGGACAGAGGCGTAAGATGG	GTCTTGCGGCGTGGCTTGTA
*hsd3b7*	GGATTCCTCGGCAGACACCTACT	GTCCGCTCTGTGCTGAGTTCATT
*Foxh1*	GGCAGTGGAGTTGAGTCGTGTTC	GTGGCTCCGATTCTGGCTTGTG
*foxr1*	AGCCTCAAAGTCCAGCAGATTTACC	AGGTTGTGTCGGATGGTGTTCTTC
*hsd17b12a*	TCCTCAACATCTCTTCTGCCAGTG	TGACGCCTGTACTCCTCCTGAA
*hsd17b10*	TGAAGCGAGGCATCGGAACATC	AACGGCGGTGGAAGAAGAAGAAG
*zp3b*	TGCGGAACTCAACTCTCATCAACAA	AATGGCATCCACAGCATACCTCTT
*zp3d.2*	GCGACGAACAGAGACTACCAGAGA	TGCTCACCTTCCACCTCAATCCA
*sox11*	ACGAAGAAGTGCGGAACAACAACA	TGCTGGAGGAGGAGGAGGATGT
*β-actin*	CAGCACACCGATGGAGACAGATG	ATGCCATTCTTGAGCGGAGACAT

**Table 2:** Screening of differentially expressed genes in the gonads of male and female specimens of Nao-zhou stock large yellow croaker.

Gonad	Symbol	Log_2_(FC)	Description
Ovary	zp2l2	11.14630802	zona pellucida sperm-binding protein 4
	gdf9	11.05705205	growth differentiation factor 9
	zp3c	10.70730617	zona pellucida sperm-binding protein 3
	zar1l	10.62065522	zygote arrest 1 like
	sox19b	10.1033617	transcription factor Sox-19b-like
	zar1	8.386104813	zygote arrest 1
	hsd17b1	7.970755402	hydroxysteroid 17-beta dehydrogenase 1
	hsd17b12a	7.970755402	hydroxysteroid 17-beta dehydrogenase 12
	zp3d.2	7.81913224	zona pellucida sperm-binding protein 3
	foxr1	7.702006538	forkhead box protein N5
	zp3b	7.185110583	zona pellucida sperm-binding protein 3
	foxh1	5.586284908	forkhead box protein H1
	figla	5.203460558	factor in the germline alpha
	igf2bp3	4.574750365	insulin like growth factor 2 mRNA binding protein 3
	FOXO3	3.869227906	forkhead box protein O3
	sox8a	3.460606657	transcription factor Sox-8
	cpeb1a	3.290123152	cytoplasmic polyadenylation element binding protein 1
	sox13	3.24331826	SRY-box transcription factor 13
	CYP27A	2.982813489	sterol 26-hydroxylase, mitochondrial
	sox10	2.956931278	SRY-box transcription factor 10
	stard10	2.900182693	StAR related lipid transfer domain containing 10
	hsd17b10	2.89519422	hydroxysteroid 17-beta dehydrogenase 10
	fgf20b	2.777419716	fibroblast growth factor 20
	gdf6a	2.593524514	growth differentiation factor 6
	sox17	2.550000123	SRY-box transcription factor17
	igf2bp1	2.317251608	insulin-like growth factor 2 mRNA-binding protein 1
	fstl4	2.288907325	follistatin like 4
	cyp11a2	2.216067902	cholesterol side-chain cleavage enzyme, mitochondrial
	sox11	2.03493379	SRY-box transcription factor 11
	cyp26a1	1.916668956	cytochrome P450 26A1
	spag7	1.826827833	sperm associated antigen 7
	SOX18	1.815129653	transcription factor Sox-18B
	HDAC2	1.702780298	histone deacetylase 2
	luzp1	1.631532334	leucine zipper protein 1
	igf2b	1.562199298	insulin like growth factor 2
	hsd3b1	1.208889923	hydroxy-delta-5-steroid dehydrogenase, 3 beta- and steroid delta-isomerase 1
Testis	fgf8b	−1.044873962	fibroblast growth factor 8b
	spata20	−1.216888207	spermatogenesis associated 20
	fgf17	−1.376083865	fibroblast growth factor 17
	sox9b	−1.380135688	transcription factor Sox-9
	dmrta2	−1.409255147	DMRT like family A2
	amh	−1.556690549	anti-Mullerian hormone
	cyp19a1a	−1.62147253	aromatase-like
	nanos1	−1.826213949	nanos homolog 1
	hdac5	−1.829685137	histone deacetylase 5
	FOXN2	−1.971653054	forkhead box N2
	FOXN2	−1.971653054	forkhead box N2
	cyp11c1	−2.73085247	cytochrome P450 11B, mitochondrial
	bmper	−2.903704155	BMP binding endothelial regulator
	hdac8	−2.906206302	histone deacetylase 8
	SPAG1	−2.930619527	sperm associated antigen 1
	tdrd6	−3.451861972	tudor domain-containing protein 6
	tdrd12	−3.58760714	tudor domain-containing 12
	tdrd1	−3.58760714	tudor domain-containing 1
	tdrd7b	−3.591337951	tudor domain-containing protein 7B
	samhd1	−3.795859283	SAM and HD domain containing deoxynucleoside triphosphate triphosphohydrolase 1
	nanos2	−4.461788579	nanos homolog 2
	fgf23	−5.392317423	fibroblast growth factor 23
	spata22	−5.900464543	spermatogenesis associated 22
	dmrt2a	−6.097090013	doublesex and mab-3 related transcription factor 2
	foxl1	−6.354149184	forkhead box protein L1
	spag6	−6.42149634	sperm associated antigen 6
	dmrt3a	−6.772589504	doublesex and mab-3 related transcription factor 3
	dmrt1	−6.985173364	doublesex- and mab-3-related transcription factor 1
	wnt4b	−7.882643049	protein Wnt-4
	fstl5	−8.448460501	follistatin like 5
	fgf13b	−8.518325308	fibroblast growth factor 13
	fgfbp1b	−11.66370634	fibroblast growth factor-binding protein 1
	pdgfc	−11.75335605	platelet derived growth factor C
	spef2	−12.2123807	sperm flagellar 2

## Data Availability

The raw reads used in this article have been deposited into the Sequence Read Archive (SRA) of the NCBI database under BioProject accession number: PRJNA1173910.

## Supplementary Materials

The following supporting information can be downloaded at: https://www.mdpi.com/article/10.3390/ani14223261/s1, Table S1: Transcriptomic read quality data. The testis group includes MG-1, MG-2, and MG-3. The ovary group in-cludes FG-1, FG-2, and FG-3; Table S2: Comparison of valid sequencing data with the reference genome.
