# Supplementary material for: Comparative Transcriptome Analysis of Sexual Differentiation in Male and Female Gonads of Nao-Zhou Stock Large Yellow Croaker (Larimichthys crocea)
Source: Animals (Basel). 2024 Nov 13;14(22):3261. doi: 10.3390/ani14223261 (PMC11591422; doi:10.3390/ani14223261)
Supplement: Supplementary file 1 [file animals-14-03261-s001.zip › animals-3225160-supplementary.pdf]

Supplementary Table S1. Transcriptomic read quality data.

Sample	Raw data (bp)	Clean data (bp)	Q20 content (%)	Q30 content (%)	GC content (%)
MG-1	6,600,226,500	6,524,411,716	98.47%	95.63%	51.02%
MG-2	6,328,342,500	6,257,508,800	98.54%	95.82%	50.84%
MG-3	6,316,740,000	6,263,709,601	98.74%	96.30%	50.83%
FG-1	6,210,486,000	6,147,200,426	98.72%	96.23%	50.54%
FG-2	6,503,824,500	6,434,316,952	98.68%	96.23%	50.60%
FG-3	6,373,163,100	6,312,008,800	98.72%	96.31%	50.40%

The testis group includes MG-1, MG-2, and MG-3. The ovary group includes FG-1, FG-2, and FG-3.

Supplementary Table S2. Comparison of valid sequencing data with the reference genome.

Item	MG-1	MG-2	MG-3	FG-1	FG-2	FG-3
Total reads	41,132,626	43,027,260	42,254,882	43,510,148	41,743,618	41,795,830
Total mapped reads	35,130,819 (85.41%)	36,648,590 (85.18%)	36,266,505 (85.83%)	37,019,034 (85.08%)	35,611,974 (85.31%)	35,726,934 (85.48%)
Percent of reads mapped to exon of genome	28,489,382 (81.10%)	29,663,658 (80.94%)	29,371,731 (80.99%)	28,948,071 (78.20%)	27,856,136 (78.22%)	27,941,204 (78.21%)
